# Enhancing the Isolation and Performance of Control Planes for Fog Computing

**DOI:** 10.3390/s18103267

**Published:** 2018-09-28

**Authors:** Kyungwoon Lee, Chiyoung Lee, Cheol-Ho Hong, Chuck Yoo

**Affiliations:** 1 Department of Computer Science and Engineering, Korea University, 145 Anam-ro, Seongbuk-gu, Seoul 02841, Korea; kwlee@os.korea.ac.kr; 2 Institute of Convergence Technology, Korea Telecom Corporation, 70 Yuseong-daero 1689 beon-gil, Yuseong-gu, Daejeon 34047, Korea; chiyng.lee@kt.com; 3 School of Electrical and Electronics Engineering, Chung-Ang University, 84 Heukseok-ro, Dongjak-gu, Seoul 06974, Korea; cheolhohong@cau.ac.kr

**Keywords:** fog computing, software-defined networking, Linux network stack

## Abstract

Fog computing, which places computing resources close to IoT devices, can offer low latency data processing for IoT applications. With software-defined networking (SDN), fog computing can enable network control logics to become programmable and run on a decoupled control plane, rather than on a physical switch. Therefore, network switches are controlled via the control plane. However, existing control planes have limitations in providing isolation and high performance, which are crucial to support multi-tenancy and scalability in fog computing. In this paper, we present optimization techniques for Linux to provide isolation and high performance for the control plane of SDN. The new techniques are (1) separate execution environment (SE2), which separates the execution environments between multiple control planes, and (2) separate packet processing (SP2), which reduces the complexity of the existing network stack in Linux. We evaluate the proposed techniques on commodity hardware and show that the maximum performance of a control plane increases by four times compared to the native Linux while providing strong isolation.

## 1. Introduction

The low latency requirement of the Internet of Things (IoT) has introduced a new computing paradigm called fog computing that places a small to medium size of computation resources (e.g., compute, storage and networking elements) close to IoT devices [1,2]. Despite the massive computing power of traditional cloud computing, IoT applications can suffer from large latency when they utilize traditional cloud computing for data processing [3]. This is because cloud data centers may be distant from latency-sensitive IoT devices [4]. Fog computing resources can be accessed by IoT devices at a one-hop distance, so that they can process delay-sensitive data generated from IoT devices in a timely manner. IoT application developers can offload expensive computation tasks from their devices to fog computing [2], which bridges a distant central cloud and IoT devices [1]. Several studies on fog computing platforms have been proposed as follows: ParaDrop [5], Mobile fog [6] and Cloudlets [7].

Software-defined networking (SDN) introduces a new networking architecture that separates control planes and data planes: the former manage network switches and the later forward network packets. The SDN architecture allows network operators or cloud providers to control remote network switches with a global network view in a centralized manner. A control plane consists of an SDN controller and control applications. The SDN controller provides abstractions, essential services and common application programming interfaces (APIs) to the control applications [8,9]. Based on the information and services provided by the controller, control applications determine the behavior of data planes at runtime.

The programmable characteristic of SDN offers several advantages such as flexibility and scalability, which can satisfy application-specific requirements of fog computing such as low latency [10]. For example, SDN controllers can construct virtual networks of different tenants to isolate network traffics from each other [1] and perform load balancing to distribute traffic loads between different fog nodes to guarantee latency-sensitive data processing [2].

Both SDN controllers and control applications run as separate processes in user-space on an operating system (OS), such as Linux. Since existing OSs including Linux are not designed for SDN controllers and control applications, they face several issues as follows.

- Lack of isolation: Recent studies [11,12,13,14,15] report that rapidly developed prototype control applications can go awry. Furthermore, third-party control applications can contain unexpected vulnerabilities, fatal instabilities or even malicious logic. These malfunctioning control planes can affect other tasks running on the same physical machine. In particular, when different virtual networks are simultaneously constructed [16,17], the faulty control planes can cause the crash of the entire system, which leads to the loss of network control. This is because existing OSs run control planes as user-level processes and do not provide additional access control or an isolated execution environment. Therefore, it becomes necessary to provide strong isolation in the execution environment of control planes.
- Low performance: OSs are designed to support various applications including control planes, which offer a variety of network functions such as encryption/decryption, firewall and rate limiting. Every packet arriving at the system must go through the entire network stack of the OS before reaching the SDN controller. Moreover, incoming packets must wait to be processed in order by the corresponding SDN controller. This is due to the fact that the existing network stack processes packets one by one. Thus, incoming packets can be dropped, when packets arrive successively at high speed [18]. This can result in serious performance degradation of the SDN controller.

Previous studies [11,12,13,14,15,19,20,21,22,23] introduced several techniques to provide isolation and high performance of control planes. They mainly focused on re-designing the controller architecture. For example, Drutskoy et al. [20] utilizes container virtualization to separate multiple control applications on a shared SDN controller platform, while Shin et al. [11] develops context separation between control applications and the SDN controller. Even though these studies prevent faulty control applications from affecting the SDN controller, they cannot isolate multiple SDN controllers of different tenants. In order to assure the integrity of multiple SDN controllers, a trust-oriented controller proxy (ToCP) [12] is suggested. ToCP provides trustable network environments by inspecting control messages of different SDN controllers. While these techniques improve the degree of isolation, in return, they increase performance overhead significantly in processing control messages because they need an additional layer or components between control and data planes. This can impede latency-sensitive data processing in fog nodes, which cannot meet the low delay requirement of fog computing. In addition, re-designing the controller architecture [19,24] can improve controller performance, but has limitations because the performance bottleneck in the network stack of OSs still exists.

In this paper, we investigate how to achieve both isolation and high performance for control planes simultaneously. This paper presents an innovative approach that focuses on optimizing the OS that executes control planes rather than modifying the internal architecture of control planes. Previous studies modified the detailed operations of control planes such as how to manage control applications on SDN controllers or how to deal with control messages from network switches to improve control planes in terms of isolation and performance. However, these studies have limitations because the SDN controllers and the control applications run as user-level processes on OSs such as Linux. Therefore, the degree of isolation and the baseline of performance improvement depend on the OS running the control planes. For further enhancement of isolation and performance, this paper develops the separate execution environment (SE2) and separate packet processing (SP2), which implement the optimization techniques at the OS level, which is entirely different from existing approaches. In particular, this paper focuses on developing optimization techniques for Linux, which is the most popular OS for the deployment of control planes (Even though this paper presents optimization techniques for Linux, the fundamental approaches in this paper can be applied to other OSs such as FreeBSD and Windows). Furthermore, while developing SE2 and SP2, we maintain the semantics of Linux, such as abstracting system resources and widely-used APIs.

First, we construct SE2 to provide an isolated execution environment to a control plane. We use virtual machine (VM) abstraction so that SE2 logically isolates the resources of the control planes and avoids interference between multiple control planes concurrently running on a physical machine. Using VM abstraction has the following advantages. (1) When different control planes owned by different tenants run concurrently [25], SE2 can provide an isolated execution environment to each control plane. This enables each tenant to run its own control plane. (2) In previous studies, control planes ran as user-level processes that shared the same OS, so that control planes running simultaneously could interfere with each other. In SE2, a control plane does not access physical resources directly, and so, SE2 can also prevent the failure of a control plane from affecting others. (3) VM abstraction can improve the resource efficiency of physical resources in a data center by consolidating multiple control planes in the same physical machine.

However, VM abstraction brings the virtualization overhead to the control plane, which degrades packet processing performance. In order to minimize the virtualization overhead and improve packet processing performance, we proposes a novel packet processing routine called SP2. SP2 is a software-based optimization technique, rather than using hardware support, such as virtualization extension of special network interface cards (NICs). Other studies on high performance packet processing introduced an approach that bypasses the network stack of Linux [26,27]. However, because the bypassing approach develops new APIs for fast packet processing, existing control planes need to be modified to include the new APIs. On the other hand, SP2 maintains existing Linux APIs while achieving high performance in packet processing. SP2 reduces packet processing time by dividing the Linux network stack into two parts; a minimized poll function (MPF) that delivers received packets to kernel memory space and a protocol handler (PH) that processes TCP/IP headers. First, the MPF copies the data of received packets to a lock-free queue (LFQ) for the PH. Then, the PH invokes appropriate protocol handlers depending on the type of received packets. Using the LFQ, SP2 removes the use of spin lock in delivering the data of received packets from the MPF to the PH in order to reduce packet processing overhead. In addition, we utilize a pre-allocated reusable buffer and batching operation that reduces dynamic memory access (DMA) mapping/unmapping and memory allocation which are the major packet processing overheads.

We implement SE2 and SP2 on a Linux kernel and evaluate their benefits in terms of throughput. By reducing the overhead from virtualization of SE2, SP2 increases packet processing performance by four times compared to an existing controller running on native Linux [19].

The remainder of this paper is organized as follows. We explain existing solutions in Section 2. Section 3 presents our system design. Section 4 describes our prototype implementation. Section 5 shows the evaluation results of our prototype, and Section 6 concludes the paper.

## 2. Related Work

**Diverse SDN controllers:** SDN controllers are critical elements in the SDN architecture, which generates the network configuration for the control applications. There is a diverse set of SDN controllers in their design principles and architectural choices. Table 1 classifies existing SDN controllers with their respective architectures and characteristics. Each SDN controller can be on different programming languages, including Java, Python and Ruby, requiring different libraries, but most SDN controllers run on Linux. In addition, different SDN controllers show various performances depending on their architecture from 100 k–6000 k packets per second [9], but they are insufficient to be deployed in larger scale networks. Therefore, even though SDN brings numerous advantages from its flexibility, it is critical to improve the performance [28], especially control planes.

**Support for multiple control planes:** As control planes become diverse, research on how to support multiple control planes has become important. FlowN [20] provides a container-based tenant logic to control each virtual network, which consolidates multiple control applications. It creates virtual networks by using a database storage system to maintain a physical-virtual mapping for network elements. Tenants can share an SDN controller, and the control applications of each tenant are isolated from each other using an abstraction called container. However, because control applications must be embedded in the FlowN controller, a tenant is constrained to develop the control application using the FlowN framework. Similar to FlowN, Sasaki et al. [44] adopted a container virtualization technique to provide isolation between control planes, which executes a control plane in a container that runs as a user-level process. Even though the container-based architecture enables running multiple control planes concurrently, it cannot entirely prevent control planes from affecting each other. This is because multiple containers running concurrently share the same OS such as Linux. For example, when a container performs packet transmission, Linux disables *softirq*, which is a part of the interrupt processing [45]. As a result, other containers have to wait in order to receive packets that are processed in *softirq* until the prior packet transmission of the container is finished. On the other hand, previous work [46] allows multiple controllers based on different platforms to cooperate on managing the same shared traffic by compiling different policies in an incremental manner. However, CoVisor does not consider interference among controllers in terms of computing resources and performance, which can cause severe performance degradation or security issues.

**Lack of robustness and security in a control plane:** With the increasing number of SDN controllers and control applications, the robustness and security of existing SDN controllers and control applications are of concern owing to unexpected vulnerabilities, fatal instabilities or malicious logic [11,23]. Because control planes are made of software, bugs are, in a sense, inevitable. When a buggy control application or SDN controller performs a faulty action and crashes, it can affect other SDN controllers or control applications in the same physical machine, leading to a loss of network control. Fixing bugs of control planes is time consuming and difficult, especially in a distributed environment [13]. Moreover, there is a critical risk that a malicious user will take over the network control by exploiting the programmability of control planes using the SDN API or tampering with control applications running on the SDN controller [12].

Previous research [11,12,23] presented solutions to improve the robustness and security of controllers. Betge et al. [12] presented a trusted execution environment for SDN controllers to prevent a malicious user from manipulating the control plane. The trusted execution environment in [12] utilized additional controllers and a network hypervisor to inspect control messages of control planes. However, this requires extra resources such as additional controllers and a network hypervisor, which brings additional performance overhead from 20–30%. Therefore, this paper aims to prevent a faulty control plane from affecting other tasks with minimal resource consumption and performance overhead.

**Performance improvement of a control plane:** Tootoonchian et al. [19] pointed out that the performance of existing SDN controllers is not sufficient for deployment in an actual network environment. Therefore, they presented a multi-threaded SDN controller that handles up to 1.2 million requests per second. Even though they showed impressive performance improvement by optimizing the SDN controller itself, their system does not fully support the peak load of control packets in a large network [47]. Wang et al. [48] proposed a flow re-directing technique to minimize the response time of control planes in data centers. However, flow re-directing imposes additional burdens on the network devices when it is applied to fog networking. For example, flow re-directing requires periodic information updates in all network devices. Because fog networking includes various types of network devices such as resource-constraint gateways, the additional operation for the information update can affect the packet delivery performance. Some studies modify the OpenFlow control message operation to reduce flow setup latency and boost network throughput [24] or utilize hardware, such as a multi-core architecture [49] or a GPU [22]. However, those studies did not resolve the performance bottleneck in Linux, which limits the baseline of performance improvement. Our optimization techniques improve the performance baseline even better by optimizing the network stack in Linux, and so, other techniques for SDN controllers can benefit from our techniques.

Previous studies developed techniques to overcome the limitations of existing SDN controllers in terms of isolation and performance. However, they focused on enhancing the internal architecture of SDN controllers or introducing additional components, which still limits the degree of isolation and performance. In terms of isolation, existing techniques are still implemented as user-level processes. As SDN controllers running on the same physical server share the same OS such as Linux, a misbehaving controller can affect others quite easily. With regard to performance, because major performance bottlenecks in the network stack of the OS were not resolved in the previous work, their improvement is limited by complex network processing of the existing OS.

We aim to overcome the limitations of control planes running as user-level processes by developing two optimization techniques for Linux: SE2 and SP2. SE2 provides stronger isolation between control planes using virtual machine abstraction, which offers an independent Linux environment to each control plane in order to prevent a controller from affecting other control planes. Furthermore, SP2 revises the existing network stack of Linux and offers faster packet processing for control planes.

## 3. Design

This paper presents two optimization techniques for Linux specifically to enhance the isolation and performance of control planes in SDN. In this section, we describe the design goals of our approaches and explain the details of the proposed techniques.

### 3.1. Design Goals

In developing optimization techniques, we focus on three principal goals as follows.

- Running existing SDN controllers and control applications without modification: Most of the SDN controllers and control applications are based primarily on Linux. To be compatible with existing SDN controllers and control applications, we maintain standard Linux APIs.
- Providing isolated execution environments while removing dependency on specific hardware: To be used in an existing SDN deployment, we provide a general execution environment architecture, which is not dependent on specific hardware. In addition, the control plane in the execution environment does not affect other control planes, when multiple control planes are co-located in the same physical server.
- Guaranteeing high control plane performance compared to existing Linux: Even though several studies improved the control plane performance, they focused on optimizing the control plane itself, leaving room for further improvement: the Linux kernel. We aim to achieve high performance of control planes during packet processing by optimizing the network stack of Linux.

By satisfying three design goals, SE2 and SP2 can improve the deployment and the network performance of fog computing. First, SE2 allows different types of networks in the fog such as 3G, LTE and WiFi to be managed simultaneously in an independent manner by running multiple controllers in each isolated environment. In addition, when virtual networks interconnect geographically-dispersed fog clouds, SE2 can offer different execution environments to the control plane of each virtual network. This enables the tenants of different virtual networks in the fog to control their own virtual network independently of other virtual networks. For example, when a smart grid management application and a smart lighting application construct their own virtual networks individually, SE2 allows the two virtual networks to be managed independently by employing their different control planes in each isolated environment. Second, the high performance of control planes can result in low service latency in fog computing. Because the service latency is determined by network delay, it is important to deliver data from IoT devices to a destination as fast as possible. When a switch receives a packet of a service, the switch asks the control plane where the packet should be sent. The packet remains in the switch until the switch receives the corresponding answer from the control plane, which increases the service latency. SP2 improves the performance of existing control planes, which can reduce the service latency in fog computing.

### 3.2. SE2: Isolated Execution Environment

To provide an isolated execution environment to control planes, we develop SE2 using VM abstraction. VM abstraction provides a separate execution environment compared to process abstraction because each VM runs in its own hardware protection domain, which provides strong isolation between VMs [50]. VM abstraction separates the memory space of each control plane and provides each VM with the separate memory address space. The actual memory address of each control plane in the VM abstraction cannot be accessed by another control plane or external users (In general, the physical address of a process can be identified by using virtual-physical memory mapping in process abstraction. However, the physical address of the control plane in VM abstraction should be translated into the actual machine address by using a shadow page or hardware assistance. Therefore, it is difficult to identify the physical address in VM abstraction without the help of the hypervisor.).

Let us suppose that two tenants A and B have their own virtual networks [51] and run their control planes in different execution environments provided by SE2. When the control plane of Tenant A crashes due to malformed control packets, it should not affect the network control of Tenant B. An advantage of using VMs is that VMs are managed by a hypervisor, and if the hypervisor proves to be trustworthy, control planes in VM abstraction can be protected from malicious attacks. Moreover, reports on common vulnerabilities and exposures (CVE) [52] indicate that the number of vulnerabilities of Xen, a representative open source hypervisor, is much smaller than that of Linux. This shows that Linux is very prone to be compromised or to be offended by an attack because of its complex architecture and large code size.

Though VM abstraction offers strong isolation between multiple control planes, it also brings performance overhead in network processing. This is because the driver domain intervenes in network processing of VMs [53]. The driver domain has privileged access to the device hardware, so it delivers I/O requests of VMs, such as packet transmission and reception, to the corresponding hardware. Because of additional memory copies and context switching between the driver domain and VMs, packet processing requires significant CPU cycles in VM abstraction. As a result, the network performance of a VM environment degrades by up to 65% compared to a non-virtualized environment [54].

In order to overcome the performance issue of VM abstraction, the single root I/O virtualization (SR-IOV) [55] technology is utilized in SE2. SR-IOV is a part of the Peripheral Component Interconnect (PCI) specification, which allows VMs to access the device hardware directly without the driver domain. We configure each VM to have a virtual NIC (vNIC) per control plane with SR-IOV, and each vNIC serves as an independent path from a VM to the physical NIC and vice versa. A goal of our SE2 design with SR-IOV is to eliminate the packet delivery delay caused by VM abstraction. In addition, SE2 can enforce isolation of control planes by adopting SR-IOV, because packets of each control plane bypass the network stack of the driver domain [56].

**Figure 1:**
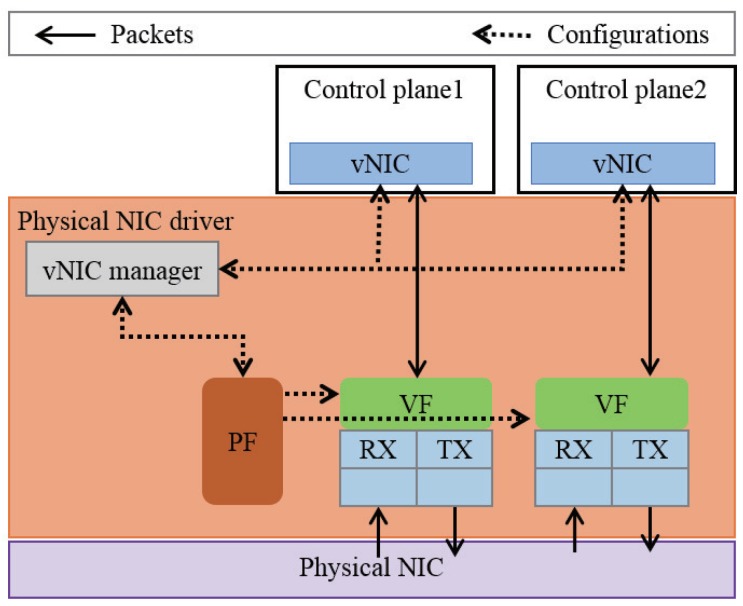
The separate execution environment (SE2) is enabled by the virtual network interface card (vNIC) manager that assigns a virtual function (VF) to each control plane. PF, physical function.

Figure 1 shows the architecture of SE2. The dotted line is the route that is used to set the network environment, and the black solid line shows the route for the data. A physical NIC has one physical function (PF) and multiple virtual functions (VFs), up to 64. The PF is a PCI function of a physical NIC that supports the SR-IOV capability. The vNIC manager in the physical NIC driver is responsible for configuring and offering VFs. A VF is associated with the PF on the physical NIC and represents a virtualized network interface of the physical NIC. SE2 utilizes VFs and assigns a VF to a control planes in the form of a vNIC. The creation and setting of a VF must be carried out by the virtual NIC manager to protect the vNIC from unauthorized access. The vNIC of a control plane sends and receives packets directly from the reception (RX) and transmission (TX) queues of the corresponding VF. For instance, when a control plane transmits a packet, it puts the packet in the TX queue of the VF through its vNIC. In contrast, when a control plane receives a packet, the packet is directly delivered to the corresponding RX queue of the control planes’ VF. As a result, in the SE2 architecture with SR-IOV, virtualization overhead from using VM abstraction is minimized by providing direct access to packet transmission and reception hardware.

SE2 can provide performance isolation between control planes by configuring the transmission rate of each VF individually. When Tenant A needs to handle a larger number of control packets compared to Tenant B, SE2 can provide different performance to each tenant by assigning a higher transmission rate to Tenant A than Tenant B. Using SE2, an administrator can manage resource allocation according to the purpose and dynamic traffic load of different control planes.

### 3.3. Separate Packet Processing

It is quite well known that the Linux network stack impairs the network performance due to unnecessary protocol handling and memory management overheads inside the kernel itself [57]. This degrades the packet processing performance of the control plane running on Linux. The reason for the low packet processing performance is that all control packets must go through the network stack in Linux before arriving at the corresponding control plane. When a control plane receives a request from a data plane, it is important to respond to the request quickly. For example, when a data plane sends a request to a control plane for routing information about a newly arrived packet, it cannot process the packet until receiving the corresponding reply from the control plane.

Previous research [18,26,27] aimed to solve the performance issue of Linux through bypassing the network stack of Linux in packet processing. By allowing user-level processes to access physical network interfaces directly, they accelerate packet processing performance dramatically. However, they require several dedicated physical cores for polling in order to process incoming/outgoing packets [16,58]. In addition, existing techniques based on bypassing the Linux kernel cause control planes’ interference with each other, because they utilize a large memory pool, which is shared by all processes and VMs running on the same physical machine [59]. For example, when a packet for a control plane is received in the memory pool, it can be manipulated by other control planes because every control plane on the physical machine has access to the memory pool.

Different from existing approaches that bypass the network stack of Linux, we do not change the fundamental semantics of Linux, but only optimize the network stack of Linux for performance improvement. We develop SP2, which divides complex packet processing into two stages in order to reduce packet processing time in Linux. By optimizing the packet processing routine of Linux, we improve the performance of control planes while preventing control planes from affecting each other. The rest of this section explains the original network processing routine of Linux in detail and presents how the routine is optimized in SP2.

**Figure 2:**
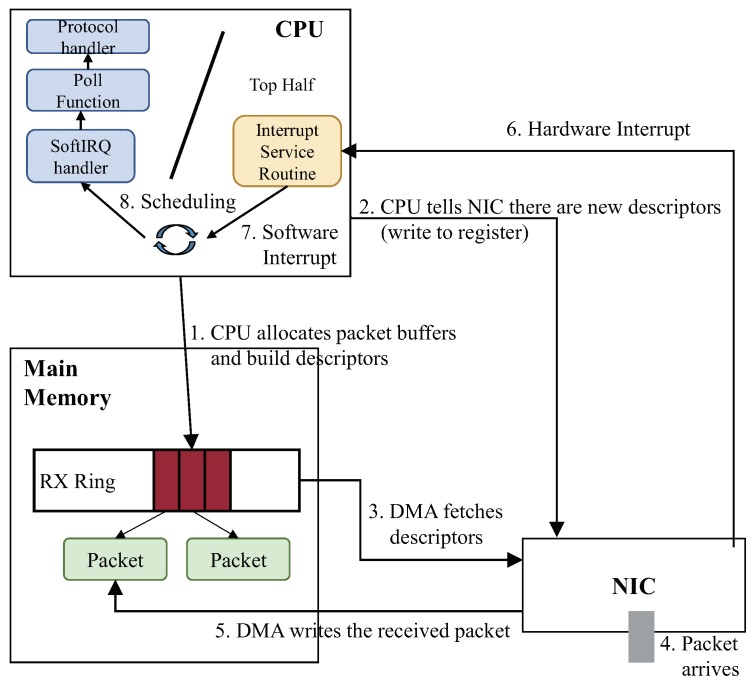
Network processing routines in Linux consist of dynamic memory access (DMA) and CPU operations.

The packet processing routine in Linux consists of dynamic memory access (DMA) operations and CPU operations, as depicted in Figure 2. At initialization, Linux allocates buffers (RX and TX Rings) to store packet descriptors for RX and TX ((1) in Figure 2). Then, Linux notifies the NIC of the allocated descriptors by writing the addresses of the descriptors into registers in the NIC (2); and DMA fetches new descriptors (3). When a packet arrives in the NIC (4), DMA writes the received packet in the memory space connected to the fetched descriptor (5). The NIC generates a hardware interrupt for packet reception after the write operation of DMA (6). By this hardware interrupt, Linux generates a software interrupt, and its corresponding software interrupt handler (*softirq*) is called.

For the efficiency of handling interrupts, an existing interrupt handler is divided into two routines called top half and bottom half. The top half includes an interrupt service routine (ISR) that deals with the hardware interrupt generated by the NIC. It only generates a software interrupt, and the top half is then terminated. Afterward, the bottom half starts by calling the *softirq* handler, which is a kernel thread called *ksoftirqd*. The bottom half is responsible for connecting the descriptor of a packet to its associated socket buffer structure, and it then calls protocol handlers (e.g., MAC, IP and TCP protocol handlers). The separate interrupt processing reduces the processing delay of hardware interrupts through fast ISR processing of the top half.

However, when a control plane receives packets at high speed, incoming packets can be dropped because of the long packet process routine in the bottom half. This is because when the speed of incoming packets is faster than the packet processing time, the RX buffers in the NIC become full and cannot store more incoming packets. If a request from data planes gets dropped, the data planes cannot receive necessary information such as routing information. This results in the loss of network control and affects data processing in fog nodes.

**Figure 3:**
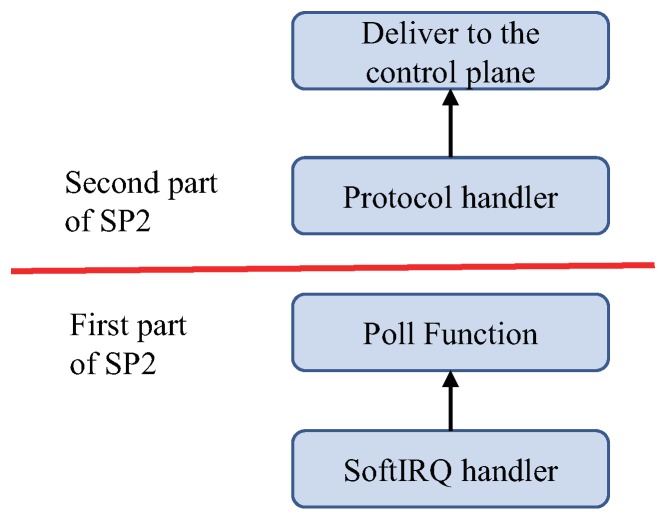
Separate packet processing (SP2) divides network processing routines into two stages, a minimized poll function (first part) and a protocol handler (second part).

To overcome the limitation of the existing packet processing routine in Linux, we develop SP2, separate packet processing. Different from the bottom half that processes incoming packets one by one in a single path, SP2 handles packets in two stages with batching. First, SP2 divides the bottom half into two parts; a minimized poll function (MPF) and a protocol handler (PH) as depicted in Figure 3. The MPF and the PH run as an individual kernel thread; the MPF is run in the *ksoftirqd* kernel thread, and the PH is run in a new thread called the SP2 thread. Then, SP2 utilizes a lock-free queue (LFQ) for transferring the data of incoming packets between *ksoftirqd* and the SP2 thread.

The MPF only copies incoming packets continuously to the LFQ for the PH. The LFQ gets rid of unnecessary spin lock overhead when the MPF transfers the incoming packets to the PH. Moreover, incoming packets do not get dropped because the MPF keeps moving incoming packets from the RX buffers in the NIC to the LFQ. The PH performs packet processing including checksum validation and firewalling and delivers the packets to the control plane. When the PH delivers the packets to the control plane, it does not copy the packets one by one. When packets are bound to the same control plane, the PH copies multiple packets at once. Furthermore, we exploit the multi-core architecture of modern servers by allocating separate CPU cores to each of the MPF and the PH. Even though the MPF and the PH run in different CPU cores in parallel, they do not require lock operations for transferring packets. This is because the MPF and the PH work as a single-producer and a single consumer, respectively. Through the separation of the bottom half process, SP2 reduces packet processing time in Linux and prevents performance degradation of the control plane.

**Figure 4:**
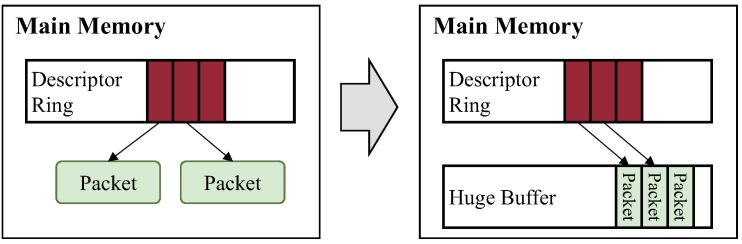
The huge reusable buffer allocates a memory pool for incoming packets at the initialization of SP2.

Reusable huge buffer: In addition to the separation of the packet processing routine, we adopt a huge reusable buffer, which is a technique used for high speed packet processing. This reduces overhead for memory allocation in packet processing, as depicted in Figure 4. Different from previous studies [18,26], SP2 does not allocate a huge buffer for every process on a server. A huge reusable buffer is assigned to each control plane individually in order not to allow a control plane to have access to the huge buffer of another control plane.

In Linux, every time each packet is received or transmitted, memory allocation overhead is caused by DMA. Every time a control plane receives a packet, a DMA operation for copying the packet from the device to main memory is required. Then, a page for the packet is allocated in main memory, and the mapping function is called to translate the virtual address for the page to its physical address. The physical address is then sent to DMA. After DMA finishes copying, the page is unmapped from DMA and de-allocated. This increases packet processing time of control planes and brings severe performance degradation when incoming packets arrive at high speed. This is because the DMA operation is performed repeatedly for every incoming packet [18]. In order to reduce the overhead from a DMA mapping/unmapping operation, SP2 enables a vNIC to allocate a huge reusable buffer in the VM abstraction and to call the mapping function for DMA at initialization of the driver.

Our huge reusable buffer consists of a ring buffer with 65,536 entries. The size of each entry is 1500 bytes, which is sufficiently large to store one Ethernet packet. The mapping function delivers the start address and the total length of the buffer to DMA. DMA then writes and reads data from the start address of the buffer to its end. After all data in the buffer are written and read, the buffer is not de-allocated and used continuously for other DMA requests until the system is terminated. This eliminates memory allocation and mapping overhead for DMA by minimizing the number of function calls.

## 4. Implementation

**Figure 5:**
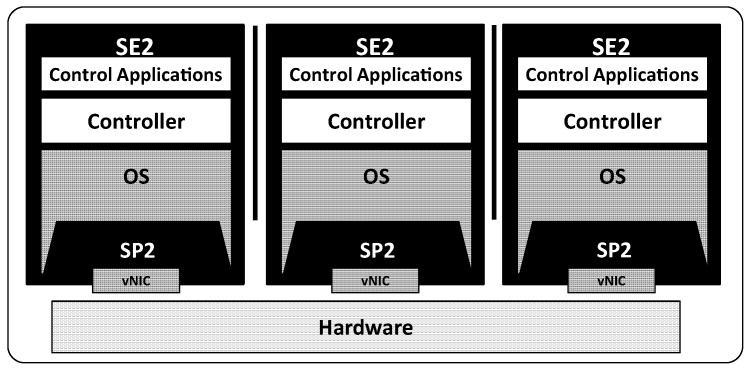
Each control plane consists of an independent SE2 and SP2.

The implementation environment is configured as follows: SE2 is constructed as a virtual machine (VM) utilizing a kernel image of Linux 2.6.32 on Xen hypervisor 4.0. At the initialization of SE2, a virtual network device (vNIC) is assigned to the VM by the virtual NIC manager in the network driver, ixgbe-3.17.3. Multiple vNICs of different VMs can be mapped to a physical NIC simultaneously. Each vNIC has its own MAC address, and incoming packets are delivered to corresponding control planes directly by the MAC addresses. When the initialization of SE2 finishes, a tenant can run a control plane that consists of a controller and control applications on the VM as in Figure 5.

We modify the ixgbevf-2.12 driver and develop an additional kernel module (SP2 module) to implement SP2, which handles incoming packets from a vNIC in a control plane. At the initialization of the ixgbevf driver, the driver allocates a huge reusable buffer that stores incoming packets. The buffer is mapped to direct memory access (DMA) and does not require an additional mapping/unmapping operation because the buffer is re-used for the next incoming packets after the packet in the buffer is delivered to the SP2 module.

Followed by the initialization of the ixgbevf driver, the SP2 module allocates a lock-free queue to retrieve packets from the huge reusable buffer. Furthermore, the SP2 module generates a kernel thread, a SP2 thread, that performs additional packet processing such as IP header checksum validation and firewalling. To divide the packet reception procedure into the two parts (the MPF and the PH) in SP2, we modify the receive function of the ixgbevf driver (i.e., ixgbevf_clean_rx_irq).

The minimized poll function (MPF) performs the first part of SP2. The MPF in the ixgbevf driver just keeps fetching packets from the huge reusable buffer, which are delivered to the lock-free queue in the SP2 module. The MPF can deliver the incoming packets in the vNIC to the lock-free queue immediately without waiting for protocol processing of prior packets. This reduces packet processing time in the ixgbevf driver and allows the driver to handle more incoming packets.

When the number of fetched packets in the lock-free queue of the SP2 module exceeds a batch size, the SP2 module wakes up the SP2 thread to execute the protocol handler (PH) as depicted in Figure 3 of the revised manuscript. The SP2 thread checks the source/destination IP addresses in order to identify whether the packet is bound for the control plane. If the packet is for the control plane, the SP2 thread runs an IP protocol handler. This IP protocol handler conducts the same operation as the Linux network stack. However, it is more efficient than that of Linux, because the IP protocol handler performs header checksum validation and firewalling only once for the first packet out of the incoming packets having the same source/destination IP addresses. When the PH finishes, SP2 copies the packets of the batch size to the control plane at once.

## 5. Evaluation

In this section, we first evaluate how much performance improvement is made by SE2 and SP2, respectively. Then, we present a performance result when multiple control planes run concurrently with both SE2 and SP2. To observe the baseline performance of Linux regardless of the type of the SDN controller or control applications, we evaluate the performance of the network stack without SDN controllers and control applications. We use two physical servers that have six-core dual processors with an X8DAH+ main board and 12 GB of memory and connect the servers with 10 Gigabit Ethernet. For SR-IOV, we utilize three Intel 10-Gbps 82599 NICs, each of which has two ports. We assign two CPUs, 2 GB of memory, and two RX/TX queues on a physical NIC interface to each control plane.

In our evaluation, we run multiple control planes concurrently up to five. This is because three or four controllers was enough to reduce the average network latency when the network topology consists of 41 edges [60]. Therefore, the maximum five control planes are sufficient to support the fog constructed using 40 servers at most [61]. In addition, we configure packet sizes ranging from 64 bytes-1500 bytes. This shows that the control planes with our SE2 and SP2 can support various control messages where the size of control messages varies depending on the type of requests from fog network devices [62].

### 5.1. SE2

**Figure 6:**
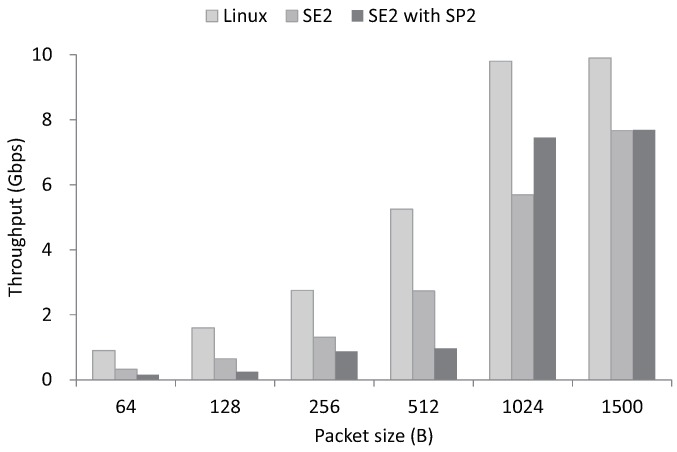
SP2 minimizes the performance overhead of SE2 from adopting VM abstraction, which achieves 80% of native performance with packets larger than 1024 bytes.

In SE2, we adopt VM abstraction and SR-IOV to provide an isolated execution environment to control planes. We measure the packet processing performance of SE2 and compare the result with Linux. The packet sizes for the evaluation range from 64 bytes–1500 bytes, and a single core is utilized for the packet processing. In the evaluation, both Linux and SE2 performs packet reception. Furthermore, we measure the packet processing performance when we run SE2 with SP2, which performs the IP protocol handler. As illustrated in Figure 6, SE2 achieves 50% of native performance when it only performs packet reception. Even with SR-IOV, SE2 shows lower performance than the non-virtualized environment. When we run SE2 with SP2, SE2 achieves almost 80% of native performance in packets larger than 1024 bytes, which shows that SP2 effectively reduces the packet processing overhead of Linux.

### 5.2. SP2

We evaluate how much performance improvement is gained by SP2, compared to Linux. We measure the packet processing rate of Linux and SP2 when Linux and SP2 performs IP forwarding. In this evaluation, we generate incoming traffic using 64 and 1500 bytes packets to maximize the processing load on the CPU and NIC, respectively. As shown in Table 2, SP2 increases the packet processing rate by two times compared to Linux. This is because SP2 handles incoming packets using two different kernel threads: *ksoftirqd* for the MPF and the SP2 thread for the PH.

**Figure 7:**
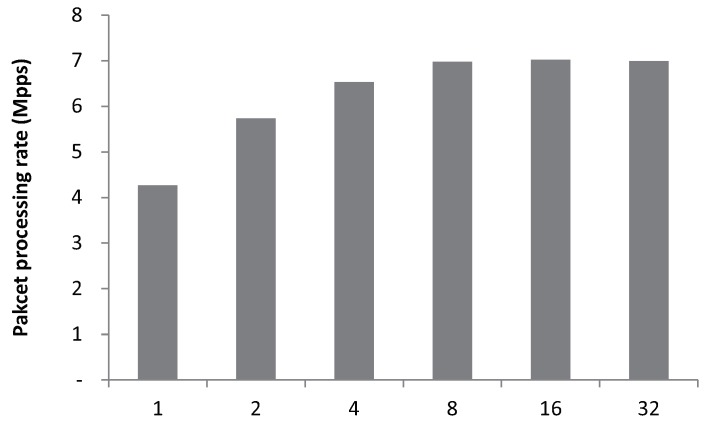
Packet processing performance of SP2 increases as the batch size increases from 1–8. When the batch size exceeds eight, there is no further performance improvement.

In addition, we measure control plane performance when we configure different batch sizes in the huge reusable buffer. Batch sizes determine how many packets having the same source/destination addresses will be moved from the MPF to the PH. As depicted in Figure 7, when we increase the batch size from 1–32, we achieve the highest throughput at eight. This is because the larger batch size results in a longer time for the memory copy between the huge reusable buffer and the LFQ. Even though SP2 reduces the overhead from memory copy by using the batching operation, the larger batch size does not guarantee the higher packet processing performance.

### 5.3. SE2 + SP2

**Figure 8:**
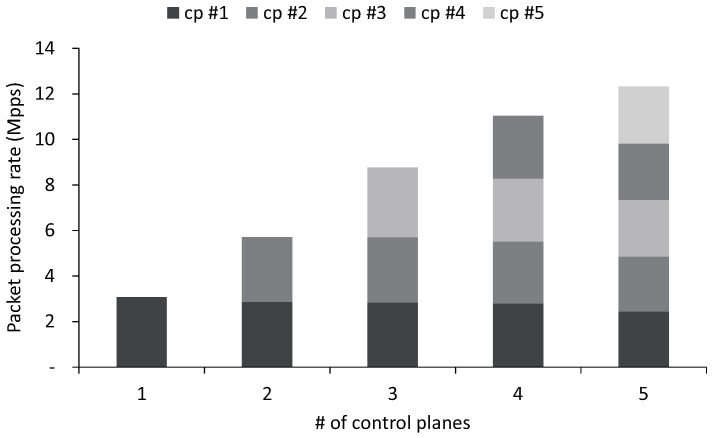
Aggregated performance with SE2 and SP2 achieves 38 Gbps when five control planes run concurrently. cp, control plane.

At last, we measure the overall performance when multiple control planes ranging from 1–5 are running simultaneously. The control planes share the same physical NIC and receive packets through the different vNICs. They transmit to the incoming 384-byte (The average control packet size varies depending on the SDN controller or the type of request; Floodlight is 510 bytes, for example. We choose 384-byte packets for our evaluation to impose processing load on both CPU and NIC) packets by performing the PH. In addition, we assign two physical cores to each control plane to measure the maximum performance of control planes without CPU contention. Figure 8 demonstrates the maximum throughput of multiple control planes with a 384-byte control packet that consumes both CPU and network resources aggressively. A control plane is able to reach 10-Gbps throughput, which saturates the 10 Gigabit Ethernet network interface. Compared to the performance of the existing SDN controller, NOX-MT, which processes about 0.7 million packets per second using two threads, our technique increases packet processing performance of SDN controllers by four times. When we increase the number of control planes running concurrently (cp #1, cp #2 …), the aggregate performance increases linearly. As the number of control planes increases, the performance of each control plane decreases slightly because of resource contention in the physical NIC. The aggregate performance achieves 38 Gbps with five control planes, which is 76% of the theoretical maximum throughput.

## 6. Conclusions

In this paper, we present optimizing techniques for Linux, SE2 and SP2, which provide isolated execution environments and high performance to control planes in SDN. We allow multiple control planes to run on a single server by adopting VM abstraction. In addition, we address the technical challenges of using VM abstraction and achieve high performance in commodity servers. We optimize the complex network stack of Linux to reduce packet processing time, which causes performance degradation of control planes. Our evaluation results show that the optimized Linux processes over three million packets per second, which almost saturates the line rate (10 Gbps) and outperforms existing SDN controllers by four times. We plan to run various SDN controllers and control applications on the optimized Linux in a fog computing environment.

## Figures and Tables

**Table 1:** SDN controllers with programming language, target operating systems, general architecture and the type of northbound API.

Name	Programming Language	Operating Systems	Architecture	Northbound API
Beacon [29]	Java	Linux	centralized multi-threaded	Ad hoc API
DISCO [30]	Java	Linux, Mac OS	distributed	REST
Floodlight [31]	Java	Linux, Mac OS	centralized multi-threaded	RESTful API
HP VANSDN [32]	Java	Linux (Ubuntu)	distributed	RESTful API
HyperFlow [33]	C++	Linux	distributed	N/A
Onix [34]	Python, C	N/A	distributed	NVP NBAPI
Maestro [35]	Java	Linux (Ubuntu 32bit)	centralized multi-threaded	Ad hoc API
Meridian [36]	Java	Linux, Mac OS	centralized multi-threaded	extensible API layer
NOX [37]	C++	Linux	centralized	Ad hoc API
NOX-MT [19]	C++	Linux	centralized multi-threaded	Ad hoc API
OpenDaylight [38]	Java	Linux	distributed	REST, RESTCONF
OSDN controller [39]	Java	OS X (Mavericks and later), Linux (Ubuntu 64 bit)	distributed	RESTful API
PANE [40]	Haskell	OS X (10.6 and up), Linux (Ubuntu)	distributed	PANE
POX [41]	Python	Windows, Mac OS, and Linux	centralized	Ad hoc API
RyuSDN controller [42]	Python	Linux	centralized multi-threaded	Ad hoc API
SMaRtLight [43]	Java	Linux	distributed	RESTful API

**Table 2:** SP2 outperforms unmodified Linux by 74% and 77% for 64-byte and 1500-byte packets, respectively.

Packet Size	64 Bytes	1500 Bytes
Unmodified Linux	374 Kpps	381 Kpps
Linux with SP2	664 Kpps	663 Kpps

## References

[1] Moreno-Vozmediano R., Montero R.S., Huedo E., Llorente I.M. (2017). Cross-site virtual network in cloud and fog computing. IEEE Cloud Comput..

[2] Hakiri A., Sellami B., Patil P., Berthou P., Gokhale A. Managing Wireless Fog Networks using Software-Defined Networking. Proceedings of the 2017 IEEE/ACS 14th International Conference on Computer Systems and Applications (AICCSA).

[3] Yi S., Li C., Li Q. A survey of fog computing: Concepts, applications and issues. Proceedings of the 2015 Workshop on Mobile Big Data.

[4] Yu W., Liang F., He X., Hatcher W.G., Lu C., Lin J., Yang X. (2017). A survey on the edge computing for the Internet of Things. IEEE Access.

[5] Willis D., Dasgupta A., Banerjee S. ParaDrop: A multi-tenant platform to dynamically install third party services on wireless gateways. Proceedings of the 9th ACM Workshop on Mobility in the Evolving Internet Architecture.

[6] Hong K., Lillethun D., Ramachandran U., Ottenwälder B., Koldehofe B. Mobile fog: A programming model for large-scale applications on the internet of things. Proceedings of the Second ACM SIGCOMM Workshop on Mobile Cloud Computing.

[7] Satyanarayanan M., Chen Z., Ha K., Hu W., Richter W., Pillai P. Cloudlets: At the leading edge of mobile-cloud convergence. Proceedings of the 2014 6th International Conference on Mobile Computing, Applications and Services (MobiCASE).

[8] Kreutz D., Ramos F.M., Verissimo P.E., Rothenberg C.E., Azodolmolky S., Uhlig S. (2015). Software-defined networking: A comprehensive survey. Proc. IEEE.

[9] Zhao Y., Iannone L., Riguidel M. On the performance of SDN controllers: A reality check. Proceedings of the 2015 IEEE Conference on Network Function Virtualization and Software Defined Network (NFV-SDN).

[10] Bera S., Misra S., Vasilakos A.V. (2017). Software-defined networking for internet of things: A survey. IEEE Int. Things J..

[11] Shin S., Song Y., Lee T., Lee S., Chung J., Porras P., Yegneswaran V., Noh J., Kang B.B. Rosemary: A robust, secure, and high-performance network operating system. Proceedings of the 2014 ACM SIGSAC Conference on Computer and Communications Security.

[12] Betgé-Brezetz S., Kamga G.B., Tazi M. Trust support for SDN controllers and virtualized network applications. Proceedings of the 2015 1st IEEE Conference on Network Softwarization (NetSoft).

[13] Scott C., Wundsam A., Raghavan B., Panda A., Or A., Lai J., Huang E., Liu Z., El-Hassany A., Whitlock S. (2015). Troubleshooting blackbox SDN control software with minimal causal sequences. ACM SIGCOMM Comput. Commun. Rev..

[14] Porras P.A., Cheung S., Fong M.W., Skinner K., Yegneswaran V. (2015). Securing the Software Defined Network Control Layer.

[15] Yoon C., Lee S., Kang H., Park T., Shin S., Yegneswaran V., Porras P., Gu G. (2017). Flow wars: Systemizing the attack surface and defenses in software-defined networks. IEEE/ACM Trans. Netw..

[16] Dalton M., Schultz D., Adriaens J., Arefin A., Gupta A., Fahs B., Rubinstein D., Zermeno E.C., Rubow E., Docauer J.A. Andromeda: Performance, Isolation, and Velocity at Scale in Cloud Network Virtualization. Proceedings of the 15th USENIX Symposium on Networked Systems Design and Implementation (NSDI).

[17] Yang G., Yu B.Y., Jeong W., Yoo C. FlowVirt: Flow Rule Virtualization for Dynamic Scalability of Programmable Network Virtualization. Proceedings of the International Conference on Cloud Computing.

[18] Rizzo L. Netmap: A novel framework for fast packet I/O. Proceedings of the 21st USENIX Security Symposium (USENIX Security 12).

[19] Tootoonchian A., Gorbunov S., Ganjali Y., Casado M., Sherwood R. On Controller Performance in Software-Defined Networks. Proceedings of the 2nd USENIX Workshop on Hot Topics in Management of Internet, Cloud, and Enterprise Networks and Services.

[20] Drutskoy D., Keller E., Rexford J. (2013). Scalable network virtualization in software-defined networks. IEEE Int. Comput..

[21] Jin X., Gossels J., Rexford J., Walker D. Covisor: A compositional hypervisor for software-defined networks. Proceedings of the 12th USENIX Symposium on Networked Systems Design and Implementation (NSDI 15).

[22] Renart E.G., Zhang E.Z., Nath B. Towards a GPU SDN controller. Proceedings of the 2015 International Conference and Workshops on Networked Systems (NetSys).

[23] Porras P., Shin S., Yegneswaran V., Fong M., Tyson M., Gu G. A security enforcement kernel for OpenFlow networks. Proceedings of the First Workshop on Hot Topics in Software Defined Networks.

[24] Luo T., Tan H.P., Quan P.C., Law Y.W., Jin J. Enhancing responsiveness and scalability for OpenFlow networks via control-message quenching. Proceedings of the 2012 International Conference on ICT Convergence (ICTC).

[25] Li Y., Dong L., Qu J., Zhang H. Multiple controller management in software defined networking. Proceedings of the 2014 IEEE Symposium on Computer Applications and Communications (SCAC).

[26] Jeong E., Woo S., Jamshed M.A., Jeong H., Ihm S., Han D., Park K. (2014). mTCP: A Highly Scalable User-Level TCP Stack for Multicore Systems. https://www.usenix.org/node/179774.

[27] Intel Data Plane Development Kit. http://dpdk.org.

[28] Gelberger A., Yemini N., Giladi R. Performance analysis of software-defined networking (SDN). Proceedings of the 2013 IEEE 21st International Symposium on Modelling, Analysis and Simulation of Computer and Telecommunication Systems.

[29] Erickson D. The beacon openflow controller. Proceedings of the Second ACM SIGCOMM Workshop on Hot Topics in Software Defined Networking.

[30] Phemius K., Bouet M., Leguay J. Disco: Distributed multi-domain SDN controllers. Proceedings of the 2014 IEEE Network Operations and Management Symposium (NOMS).

[31] Floodlight Project. http://www.projectfloodlight.org/.

[32] HP (2013). SDN Controller Architecture.

[33] Tootoonchian A., Ganjali Y. HyperFlow: A distributed control plane for OpenFlow. Proceedings of the 2010 Internet Network Management Conference on Research on Enterprise Networking.

[34] Koponen T., Casado M., Gude N., Stribling J., Poutievski L., Zhu M., Ramanathan R., Iwata Y., Inoue H., Hama T. (2010). Onix: A Distributed Control Platform for Large-Scale Production Networks.

[35] Ng E. (2010). Maestro: A System for Scalable Openflow Control.

[36] Banikazemi M., Olshefski D., Shaikh A., Tracey J., Wang G. (2013). Meridian: An SDN platform for cloud network services. IEEE Commun. Mag..

[37] Gude N., Koponen T., Pettit J., Pfaff B., Casado M., McKeown N., Shenker S. (2008). NOX: Towards an operating system for networks. ACM SIGCOMM Comput. Commun. Rev..

[38] Medved J., Varga R., Tkacik A., Gray K. Opendaylight: Towards a model-driven sdn controller architecture. In Proceeding of the IEEE International Symposium on a World of Wireless, Mobile and Multimedia Networks.

[39] Berde P., Gerola M., Hart J., Higuchi Y., Kobayashi M., Koide T., Lantz B., O’Connor B., Radoslavov P., Snow W. ONOS: Towards an open, distributed SDN OS. Proceedings of the Third Workshop on Hot Topics in Software Defined Networking.

[40] Ferguson A.D., Guha A., Liang C., Fonseca R., Krishnamurthi S. Participatory networking: An API for application control of SDNs. Proceedings of the ACM SIGCOMM 2013 Conference on SIGCOMM.

[41] McCauley M. (2012). POX. http://www.noxrepo.org/.

[42] Telegraph N., Corporation T. (2012). RYU Network Operating System. https://ryu.readthedocs.io/.

[43] Botelho F., Bessani A., Ramos F.M., Ferreira P. On the design of practical fault-tolerant SDN controllers. Proceedings of the 2014 Third European Workshop on Software Defined Networks.

[44] Sasaki T., Perrig A., Asoni D.E. Control-plane isolation and recovery for a secure SDN architecture. Proceedings of the 2016 IEEE NetSoft Conference and Workshops (NetSoft).

[45] Monitoring and Tuning the Linux Networking Stack: Sending Data. https://blog.packagecloud.io/eng/2017/02/06/monitoring-tuning-linux-networking-stack-sending-data.

[46] Farinacci D., Li T., Hanks S., Meyer D., Traina P. (2000). RFC 2784—Generic Routing Encapsulation (GRE).

[47] Benson T., Akella A., Maltz D.A. Network traffic characteristics of data centers in the wild. Proceedings of the 10th ACM SIGCOMM Conference on Internet Measurement.

[48] Wang P., Xu H., Huang L., Qian C., Wang S., Sun Y. (2018). Minimizing Controller Response Time through Flow Redirecting in SDNs. IEEE/ACM Trans. Netw..

[49] Yeganeh S., Tootoonchian A., Ganjali Y. (2013). On scalability of software-defined networking. IEEE Commun. Mag..

[50] Garfinkel T., Pfaff B., Chow J., Rosenblum M., Boneh D. Terra: A virtual machine-based platform for trusted computing. Proceedings of the Nineteenth ACM Symposium on Operating Systems Principles.

[51] Koponen T., Amidon K., Balland P., Casado M., Chanda A., Fulton B., Ganichev I., Gross J., Ingram P., Jackson E. Network virtualization in multi-tenant datacenters. Proceedings of the 11th USENIX Symposium on Networked Systems Design and Implementation (NSDI 14).

[52] CVE Details. http://www.cvedetails.com.

[53] Santos J.R., Turner Y., Janakiraman G.J., Pratt I. Bridging the Gap between Software and Hardware Techniques for I/O Virtualization. Proceedings of the USENIX Annual Technical Conference.

[54] Liao G., Guo D., Bhuyan L., King S.R. Software techniques to improve virtualized I/O performance on multi-core systems. Proceedings of the 4th ACM/IEEE Symposium on Architectures for Networking and Communications Systems.

[55] Intel (2011). PCI-SIG SR-IOV Primer: An Introduction to SR-IOV Technology.

[56] Firestone D., Putnam A., Mundkur S., Chiou D., Dabagh A., Andrewartha M., Angepat H., Bhanu V., Caulfield A., Chung E. Azure Accelerated Networking: SmartNICs in the Public Cloud. Proceedings of the 15th USENIX Symposium on Networked Systems Design and Implementation (NSDI 18).

[57] Kim J., Jang K., Lee K., Ma S., Shim J., Moon S. NBA (network balancing act): A high-performance packet processing framework for heterogeneous processors. Proceedings of the Tenth European Conference on Computer Systems.

[58] Yasukata K., Huici F., Maffione V., Lettieri G., Honda M. HyperNF: Building a high performance, high utilization and fair NFV platform. Proceedings of the 2017 Symposium on Cloud Computing.

[59] Thimmaraju K., Shastry B., Fiebig T., Hetzelt F., Seifert J.P., Feldmann A., Schmid S. Taking control of sdn-based cloud systems via the data plane. Proceedings of the Symposium on SDN Research.

[60] Heller B., Sherwood R., McKeown N. The controller placement problem. Proceedings of the First Workshop on Hot Topics in Software Defined Networks.

[61] OpenFlow Switch Specification. https://www.opennetworking.org/wp-content/uploads/2014/10/openflow-switch-v1.5.1.pdf.

[62] Bahl V. (2014). Cloudlets for Mobile Computing. https://www.microsoft.com/en-us/research/wp-content/uploads/2016/11/Cloudlets-for-Mobile-Computing.pdf.

